# Measuring acne using Coproporphyrin III, Protoporphyrin IX, and lesion-specific inflammation: an exploratory study

**DOI:** 10.1007/s00403-017-1718-3

**Published:** 2017-02-08

**Authors:** Sachin V. Patwardhan, C. Richter, A. Vogt, U. Blume-Peytavi, D. Canfield, J. Kottner

**Affiliations:** 1 Canfield Scientific Inc., 4 Wood Hollow Road, Parsippany, NJ 07054 USA; 2 Department of Dermatology and Allergy, Clinical Research Center for Hair and Skin Science, Charité-Universitätsmedizin Berlin, Berlin, Germany

**Keywords:** Coproporphyrin III, Protoporphyrin IX, Fluorescence imaging, Acne assessment, Acne inflammation

## Abstract

*Propionibacterium acnes*: (*P. acnes*) produce Porphyrins; however, fluorescence measurement of Porphyrins from Ultraviolet-A (UVA) images has failed to establish a correlation. Acne clinical research and imaging has ignored the spectral excitation-emission characteristics and the exact pattern of the Porphyrins synthesized by *P. acnes*. In this exploratory study, for the first time, the possible relationships of Coproporphyrin III (CpIII) and Protoporphyrin IX (PpIX) fluorescence as well as acne lesion-specific inflammation measurements with clinical signs of acne are investigated. Furthermore, the sensitivity of these measurements in tracking and differentiating the known treatment effects of Benzoyl Peroxide (BPO) 5%, and combination of Clindamycin + BPO are also evaluated. Comedonal and papulopustular lesions identified by investigators during a live assessment of 24 mild-to-severe acne subjects were compared with fluorescence and inflammation measurements obtained from analysis of VISIA^®^-CR images. CpIII fluorescence spots showed a strong correlation (*r* = 0.69–0.83), while PpIX fluorescence spots showed a weak correlation (*r* = 0.19–0.27) with the investigators’ comedonal lesion counts. A strong correlation was also observed between the investigators’ papulopustular lesion counts and acne lesion-specific inflammation (*r* = 0.76). Our results suggest that CpIII fluorescence and acne lesion-specific-inflammation measurement can provide objective indication of comedonal and papulopustular acne severity, respectively. Furthermore, these measurements may be more sensitive and specific in evaluating treatment effects and early signs of acne lesion progression compared to investigators’ lesion counts.

## Introduction

The pathogenesis of acne is multifactorial with four primary factors: (1) increased sebum production, (2) alterations in the keratinization process, (3) *Propionibacterium acnes* (*P. acnes*) follicular colonization, and (4) release of inflammatory mediators [5, 6, 15, 19, 27]. Excess keratin combined with sebum partially obstructs the opening of the follicle forming a microcomedo which is the beginning of comedonal lesion development [21]. Since there is no identifiable feature visible on the skin surface, the early clinical detection and evaluation of treatment effects at this stage are not possible. Acne lesions, including comedonal lesions, are colonized by *P. acnes*. These bacteria produce pro-inflammatory mediators which contribute to the development of visible papulopustular lesions [4, 5]. Traditionally, papulopustular acne lesions were considered inflammatory, while the comedonal lesions were considered non-inflammatory. However, recent data have clearly shown a role for inflammation at all stages of acne lesion development, perhaps, sub-clinically even before comedo formation [25].

In clinical research, Investigator Global Assessment (IGA) scales are widely used for assessing overall acne severity, although interrater reliability may be low. Acne manual lesion counting is another approach. It allows researchers to differentiate between papules, pustules, open comedones, and closed comedones; however, reproducibility is limited [24]. An objective measurement technique that can quantify acne severity and is sensitive and specific in tracking treatment effectiveness may, therefore, be advantageous.

Several studies have reported analysis of Porphyrins with UVA excited fluorescence images for acne evaluation, but with limited success [1, 9, 13, 14]. In general, the imaging systems combine a UVA light source with a digital colour camera to capture a fluorescence image. The fluorescence signal is evaluated based on its colour rather than its spectral nature. Furthermore, instead of using a specific filter, the UVA blocking filter within the camera is assumed to block the excitation light. These studies report measuring Porphyrin fluorescence from the red colour channel and ignore the fluorescence in green and blue colour channels. The fluorescence signal in the red channel is mostly from Protoporphyrin IX (PpIX) (emission > 630 nm), while the Porphyrin mainly produced by *P. acnes* bacteria is Coproporphyrin III (CpIII) with greenish-orange (570–630 nm) fluorescence [7, 8, 10, 14, 17, 20, 23]. The green channel fluorescence is attributed to horn (aggregated follicular corneocytes) and was suggested for evaluating comedones [9]. However, the molecular nature, composition, and spectral response of horn are unknown.

Measurement of fluorescence and acne inflammation is possible using VISIA^®^-CR images (Canfield Scientific, Inc.). VISIA-CR uses high-power xenon strobes to excite Porphyrins at their absorption peak (405 ± 10 nm) and specific spectral filters in front of the camera for separation of PpIX and CpIII fluorescence signals. For quantitative fluorescence measurement, the captured fluorescence images are corrected for non-uniform light distribution and tissue absorption using the excitation light reflectance image [16]. Acne lesion-specific inflammation (acne-spots) is detected and measured from the RBX^®^-Red images. RBX transformation [3] allows separation of haemoglobin (Red) and melanin (Brown) absorption from the cross-polarized images using a spectro-colourimetric light-tissue interaction model.

The overall research question of this explorative study was whether, and how, CpIII and PpIX fluorescence measurements and RBX-Red based acne-spot measurements are associated with clinical signs of acne. Sensitivity of these measurements in tracking and differentiating the known treatment effects of BPO 5%, and combination of Clindamycin + BPO were also investigated.

## Materials and methods

Prior to conducting any study procedures, this study was approved by the independent Ethics Committee of the State Office of Health and Social Affairs Berlin. The study was registered at the European Union Drug Regulating Authorities Clinical Trials Database (EudraCT 2013-001716-30; https://eudract.ema.europa.eu/). The study was conducted according to the principles of the Declaration of Helsinki (1996) and Good Clinical Practice Guidelines (1996).

The data presented here was generated as part of an exploratory study to investigate the efficacy and tolerability of Tyrothricin 0.1%, an Anti-microbial Peptide (AMP), compared to two established topical acne treatments, namely Benzoyl Peroxide (BPO) 5% and combination of Clindamycin + BPO. The results of this investigation are reported elsewhere [18].

In total, 24 male and female non-smoking patients aged 18–24 years with a diagnosed mild-to-severe papulopustular acne, defined as having an Investigator Global Static Assessment (IGSA) [22, 28] score of 2–4, were included after giving their written informed consent. Patients were randomized with regard to treatment and facial side allocation. Using a split-face design, 12 out of the 24 patients received Benzoyl Peroxide (BPO) 5% (Aknefug^®^Oxid mild 5%, Dr. August Wolff GmbH & Co.KG Arzneimittel, Bielefeld, Germany), and the remaining 12 patients received a combination of Clindamycin + BPO (Duac^®^Akne Gel, GlaxoSmithKline GmbH and Co. KG, Berlin, Germany) on half of their face. All 24 patients received Tyrothricin 0.1% (Tyrosur^®^Gel, Engelhard Arzneimittel GmbH and Co. KG, Niederdorfelden, Germany) on the other half of their face. Papulopustular and comedonal lesion counting was performed manually by blinded investigators through live assessment of the subjects pre-treatment (Baseline) and post-treatment (Day 12 and Day 25).

Multi-modality facial images of the subject’s frontal, left-side, and right-side views were captured at Baseline, Day 12, and Day 25 using the VISIA-CR. The captured images included the following lighting modalities: Standard White Light Image (Clinical Photograph), Standard White Flat-Lit Image (Analysis Image), Cross-Polarized Image (Diffuse Reflectance Image), Parallel-Polarized Image (Surface Reflectance Image), PpIX Fluorescence Image, CpIII Fluorescence Image, and Excitation Light Reflectance Image. During live assessment of the subjects, the imaging system allowed the investigators to annotate the locations of the identified lesions on the photographs displayed on the screen. *X*–*Y* locations of all marked lesions along with the lesion type were saved into the subject’s record.

**Fig. 1:**
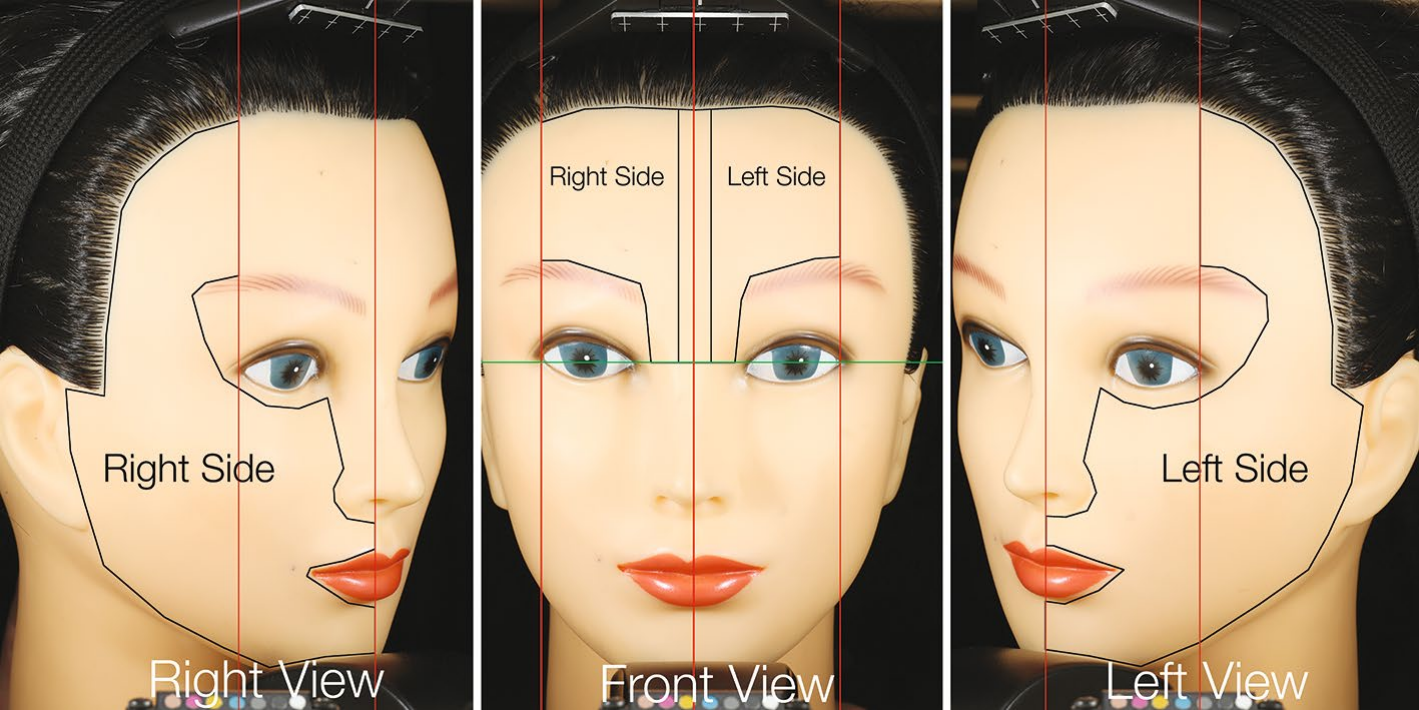
Split-face area-of-interest (AOI): As illustrated in mannequin images, the measurements for the *left-* and *right-side* views are added to the measurements from the corresponding half side of the forehead to obtain the split-face study data

All captured images were analysed while blinded to the patient’s treatment and facial side randomization, as well as the investigators’ lesion counts and lesion locations. Analysis of images started by elastically registering the multi-modality images captured during a particular session with respect to the cross-polarized image captured during that session [26]. Cross-polarized images were transformed into RBX-Red and Brown images, while the CpIII and PpIX fluorescence images were corrected for heterogeneous tissue absorption using the excitation reflectance images. Area-of-Interest (AOI) for analysis was then delineated, as illustrated in Fig. 1, using facial landmarks to include the chin, cheek, and temple regions of the face in the left- or right-side view images, and the forehead in the front-view image. The subject’s nose was not included in the AOIs. The forehead AOI was further split horizontally into left and right forehead zones.

CpIII and PpIX fluorescence spots were detected irrespective of their size and intensity from the corrected fluorescence images, while the acne-spots were detected from the RBX-Red images. For all detected features, the fractional area of the detected spots with respect to the area of the AOI was measured. For the fluorescence spots, the detection algorithm reported individual spot areas and their *X*–*Y* locations. A median fluorescence spot size was calculated for each individual subject using the Baseline measurements. The detected fluorescence spots were then filtered to retain those that were larger than a fixed pre-determined threshold.

Baseline (pre-treatment) data from all subjects were used to evaluate the relation between image analysis-based measurements and investigator assessed lesion counts. Pearson’s correlation coefficients were calculated to estimate the association of fractional areas of the fluorescence spots with the comedonal lesion counts, as well as the fractional areas of the acne-spots with the papulopustular lesion counts.

In the auto-classification technique, large fluorescence spots and acne-specific inflammation were the main features used in classifying the comedonal and papulopustular lesions, respectively [16]. Using the Baseline measurements, the association of the area of the fluorescence spots with respect to the investigators’ comedonal lesions was evaluated. For this, the total number of fluorescence spots that were larger than a fixed threshold value was compared with the investigators’ comedonal lesion counts. The range of threshold values evaluated were 60–140% of the subject’s median fluorescence spot size.

The *X*–*Y* locations of detected fluorescence spots from the subject’s images were compared with the *X*–*Y* locations of the subject’s acne lesions identified and annotated by the investigators. If a fluorescence spot was identified inside a one millimetre (1 mm) diameter circle centered at the *X*–*Y* location of the investigators’ identified lesion, it was considered a match. A percentage of comedonal and papulopustular lesions with co-located fluorescence spots were calculated for all subjects. Semantic comparison was also used to establish and verify the association between the detected spots and the investigators’ identified lesions. Similar comparison was done between the acne-spots and the subject’s acne lesions identified and annotated by the investigators.

The presence of fluorescence signal suggests *P. acnes* bacterial colonization in a follicular unit. However, during clinical evaluation, some of this bacterial activity is not accounted for due to the absence of visual skin surface indicators. This may result in lowering the sensitivity of measuring the treatment effect or delaying the evaluation. Hence, instead of using filtered fluorescence measurement, we measured the treatment effect as a percent change in the fractional area of all of the detected fluorescence spots. For similar reason, treatment effects on papulopustular lesions were measured as a percent change in the fractional area of all of the acne-spots. The percent change was measured on both Day 12 and Day 25 with respect to Baseline for the subject’s facial side treated either with BPO 5% or Clindamycin + BPO. These two measurements were compared with clinical treatment efficacy measured as a percent change in investigators’ comedonal and papulopustular lesion counts on Day 12 and Day 25 with respect to Baseline.

## Results

**Fig. 2:**
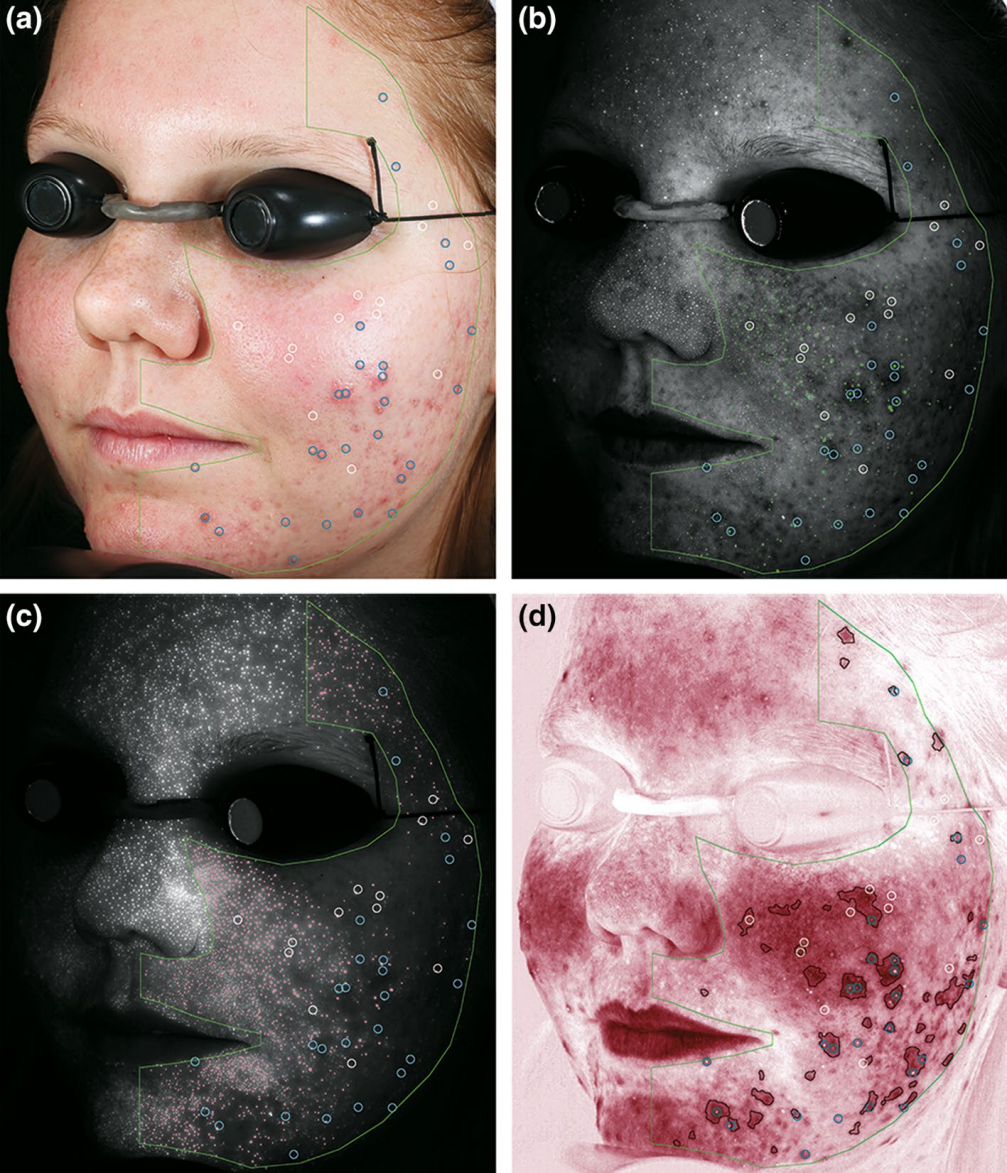
Acne lesions and associated Porphyrin fluorescence and inflammation measurements: *left* view images of a subject showing the comedonal lesions (*white circles*) and papulopustular lesions (*blue circles*) identified by the investigator on **a** standard 1 image showing the AOI (*green* outline), **b** corrected Coproporphyrin III (CpIII) fluorescence image with detected fluorescence spots (*green dots*), **c** corrected Protoporphyrin IX (PpIX) fluorescence image with detected fluorescence spots (*pink dots*), and **d** RBX-red image showing detected acne-specific inflammation (acne-spots) (*red outline*)

The lesions annotated by the investigator on the subject’s clinical image are illustrated in Fig. 2a. The association of CpIII fluorescence spots, PpIX fluorescence spots, and acne-spots with the investigator-annotated acne lesions can be seen in Fig. 2b–d, respectively.

**Fig. 3:**
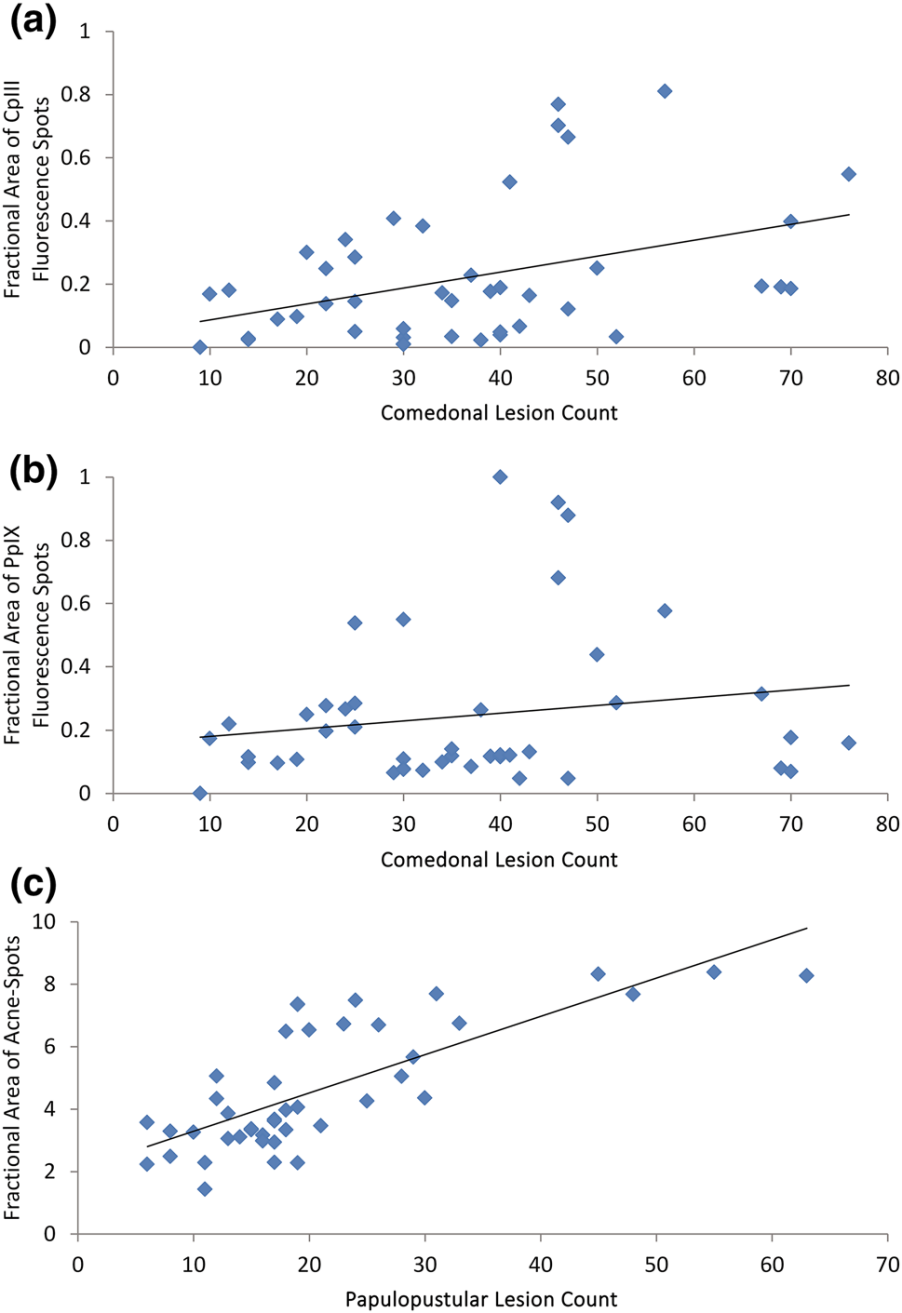
Scatter plots: **a** comedonal lesion counts versus fractional area with CpIII fluorescence spots within the AOI (*n* = 44, *r* = 0.45, *p* value < 0.01), **b** comedonal lesion counts versus fractional area with PpIX fluorescence spots within the AOI (*n* = 44, *r* = 0.21, *p* value = 0.16), and **c** papulopustular lesion counts verses fractional area of lesion-specific inflammation (*n* = 44, *r* = 0.76, *p* value < 0.001)

When comparing the investigators’ comedonal lesion counts with the number of CpIII fluorescence spots filtered using area dependent thresholds, the correlation coefficients ranged from 0.69 to 0.83. Figure 3a shows the scatter plot of the comedonal lesion counts versus the fractional area of all CpIII fluorescence spots. The correlation coefficient between the investigators’ comedonal lesion counts and the fractional area of all detected CpIII fluorescence spots was 0.45 (*n* = 44). When the locations of these fluorescence spots were compared with the locations of the investigators’ identified lesions, 94% open comedones and more than 70% of the closed comedones exhibited CpIII fluorescence signals. The intensity of the CpIII fluorescence spots was very high with a well-defined circular boundary for open comedones. For closed comedones, the CpIII fluorescence spots had a lower intensity and appeared diffused. About 20% of the papulopustular lesions also exhibited a CpIII fluorescence signal. The intensity of this signal was observed to be dependent on the haemoglobin concentration surrounding the lesions.

**Fig. 4:**
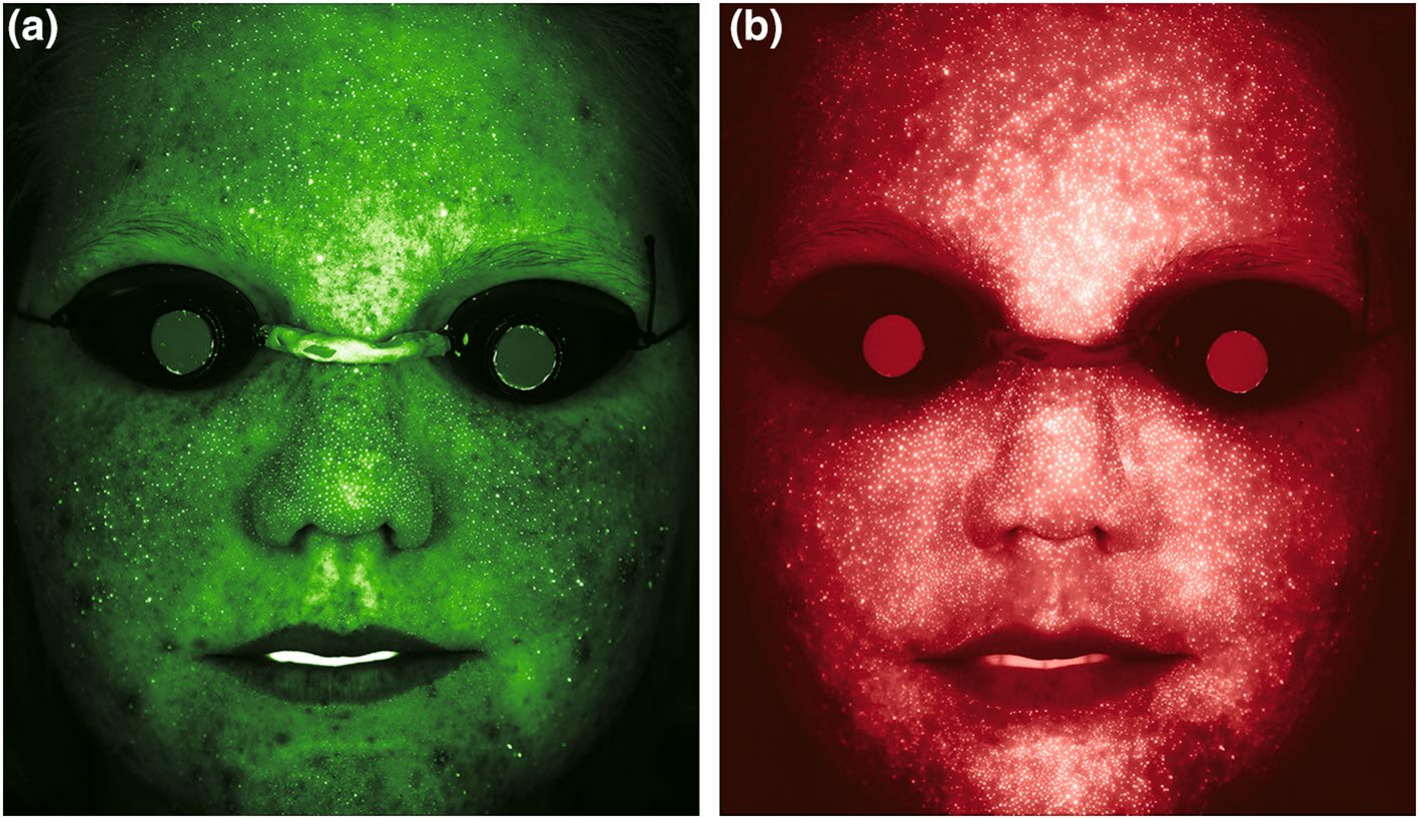
Typical facial distribution of Porphyrins: **a** CpIII fluorescence image and **b** PpIX fluorescence image of a subject show that the PpIX fluorescence spots are mainly localized in the T zone and area surrounding the nose, while the CpIII fluorescence spots can be located anywhere on the subject’s face

Figure 3b shows the scatter plot of comedonal lesion counts versus the fractional areas of all PpIX fluorescence spots. The correlation coefficient between the investigators’ comedonal lesion counts and the fractional area of all detected PpIX fluorescence spots was 0.21 (*n* = 44). When comparing the locations of these fluorescence spots with the locations of the investigators’ identified lesions, less than 15% of the comedonal lesions exhibited PpIX fluorescence signal. When comparing the comedonal lesion counts with the number of PpIX fluorescence spots filtered using area dependent thresholds, PpIX fluorescence spots showed no particular relationship with the investigators’ comedonal lesion counts. The correlation coefficients ranged between 0.19 and 0.27 for threshold values ranging 60–140% of the subject’s median fluorescence spot size. Unlike the scattered facial distribution of the CpIII fluorescence spots, visual inspection of images revealed that the PpIX fluorescence spots were mainly distributed in the T zone and in the area surrounding the subject’s nose. Figure 4 shows front-view fluorescence images of a subject illustrating the typical facial distribution of CpIII and PpIX fluorescence spots.

Figure 3c shows the scatter plot of papulopustular lesion counts versus the fractional area of detected acne-spots. The correlation coefficient between the investigators’ papulopustular lesion counts and the fractional area of acne-spots was 0.76 (*n* = 44). When comparing the locations, almost all of the investigators’ identified papulopustular lesions were located within the detected acne-spots.

**Fig. 5:**
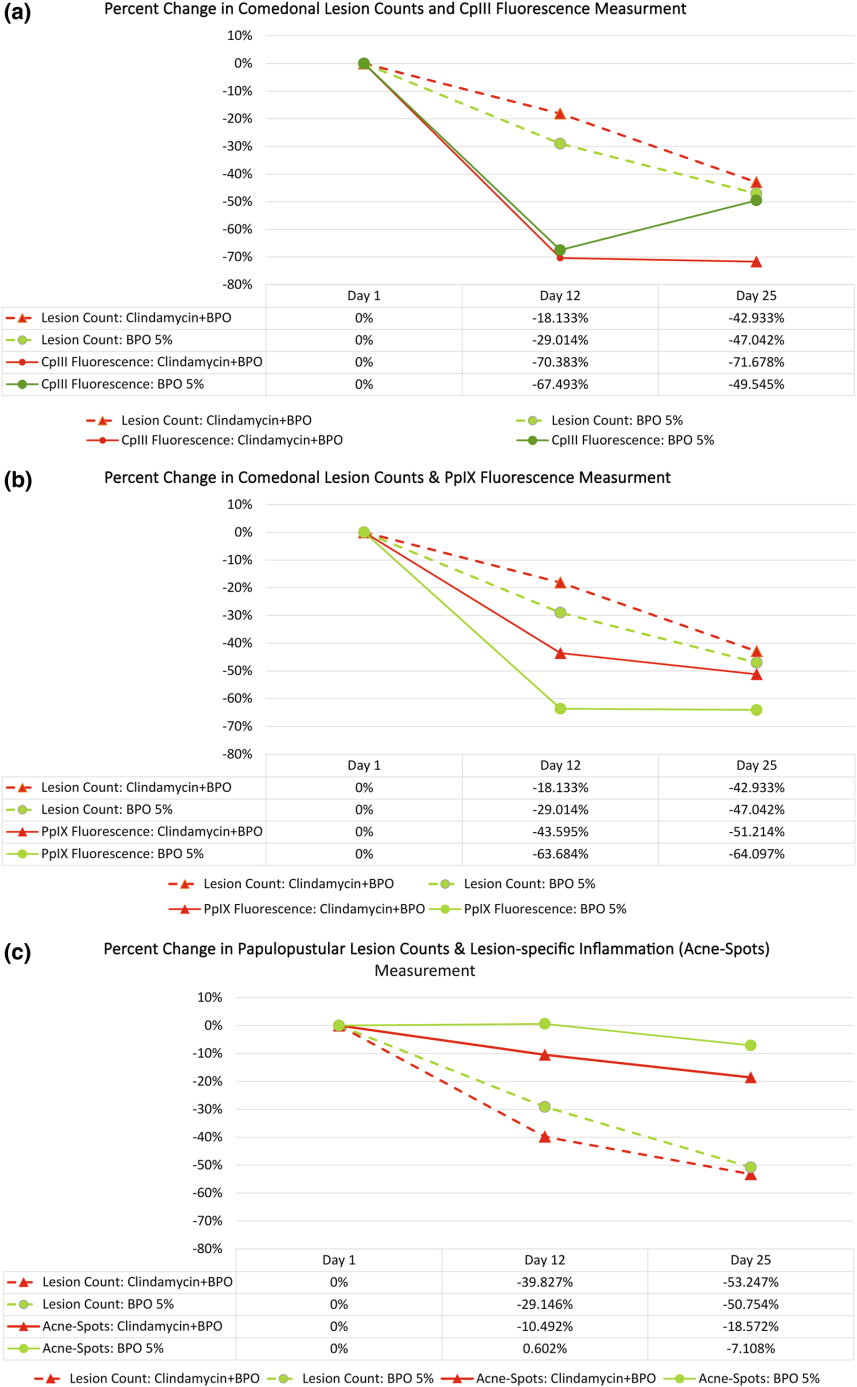
Treatment effect and its directionality: average percent change in investigators’ lesion counts and image analysis-based measurements at Day 12 and Day 25 with respect to Baseline for BPO 5% and Clindamycin + BPO treatment groups: **a** investigators’ comedonal lesion counts compared to fractional area of CpIII fluorescence spots, **b** investigators’ comedonal lesion counts compared to fractional area of PpIX fluorescence spots, and **c** investigators’ papulopustular lesion counts compared to fractional area of acne-specific inflammation (acne-spots). No significant difference in the investigators’ lesion counts was noted between the two treatment groups

Table 1 shows the mean percent change and the associated standard deviation for all the measurements at Day 12 and Day 25 with respect to Baseline for the two treatment groups. Figure 5 a, b shows the changes in the investigators’ comedonal lesion counts compared to the changes in fractional areas of CpIII and PpIX fluorescence spots, respectively. Figure 5c shows the change in the investigators’ papulopustular lesion counts compared to the change in fractional area of acne-spots.

**Table 1:** Mean percent change in acne lesion counts and fractional areas of Coproporphyrin III (CpIII)/Protoporphyrin IX (PpIX) fluorescence spots and acne-specific inflammation (acne-spots) due to treatment

Time point	Clindamycin + BPO		BPO 5%	
	Mean	SD	Mean	SD
Change in comedonal lesion count (%)				
Day 12	−18.13	46.01	−29.01	29.82
Day 25	−42.93	19.50	−47.04	27.97
Change in fractional area of CpIII fluorescence spots (%)				
Day 12	−70.38	33.73	−67.49	30.47
Day 25	−71.67	35.82	−49.54	24.30
Change in fractional area of PpIX fluorescence spots (%)				
Day 12	−43.60	15.76	−63.68	8.25
Day 25	−51.21	17.46	−64.10	7.01
Change in papulopustular lesion count (%)				
Day 12	−39.83	32.21	−29.15	23.38
Day 25	−53.25	32.39	−50.75	18.44
Change in fractional area of acne spots (%)				
Day 12	−10.50	14.63	0.60	13.80
Day 25	−18.57	9.77	−7.11	15.46

Comparison of the mean percent change (*n* = 11) and standard deviation (SD) in the fractional area of fluorescence spots and acne-spots with the percent change in the investigators’ comedonal and papulopustular lesion counts at Day 12 and Day 25 with respect to Baseline for the Clindamycin + BPO and BPO 5% treatment groups

When comparing the average percent change in the investigators’ comedonal lesion counts with respect to Baseline, at Day 12, BPO 5% showed about 10% more reduction compared to Clindamycin + BPO. However, at Day 25, no differences were observed between the two treatment groups. When comparing the average percent change in the fractional area of CpIII fluorescence spots, both treatments at Day 12 showed a reduction in the fluorescence measurement by about 70%. However, at Day 25, while Clindamycin + BPO continued to maintain almost the same amount of reduction, the reduction due to BPO 5% alone lowered to about 50%. When comparing the percent change in the fractional area of PpIX fluorescence spots, BPO 5% showed a stronger reduction compared to Clindamycin + BPO at both Day 12 and Day 25.

When comparing the average percent change in the investigators’ papulopustular lesion counts with respect to Baseline, at Day 12, Clindamycin + BPO showed about 10% more reduction compared to BPO 5%. However, at Day 25, no significant differences were observed between the two treatment groups. When comparing the percent change in the fractional area of acne-spots between the treatment groups, Clindamycin + BPO showed a stronger reduction on both Day 12 and Day 25 compared to BPO 5%, with BPO 5% showing almost no change at Day 12 with respect to Baseline.

**Fig. 6:**
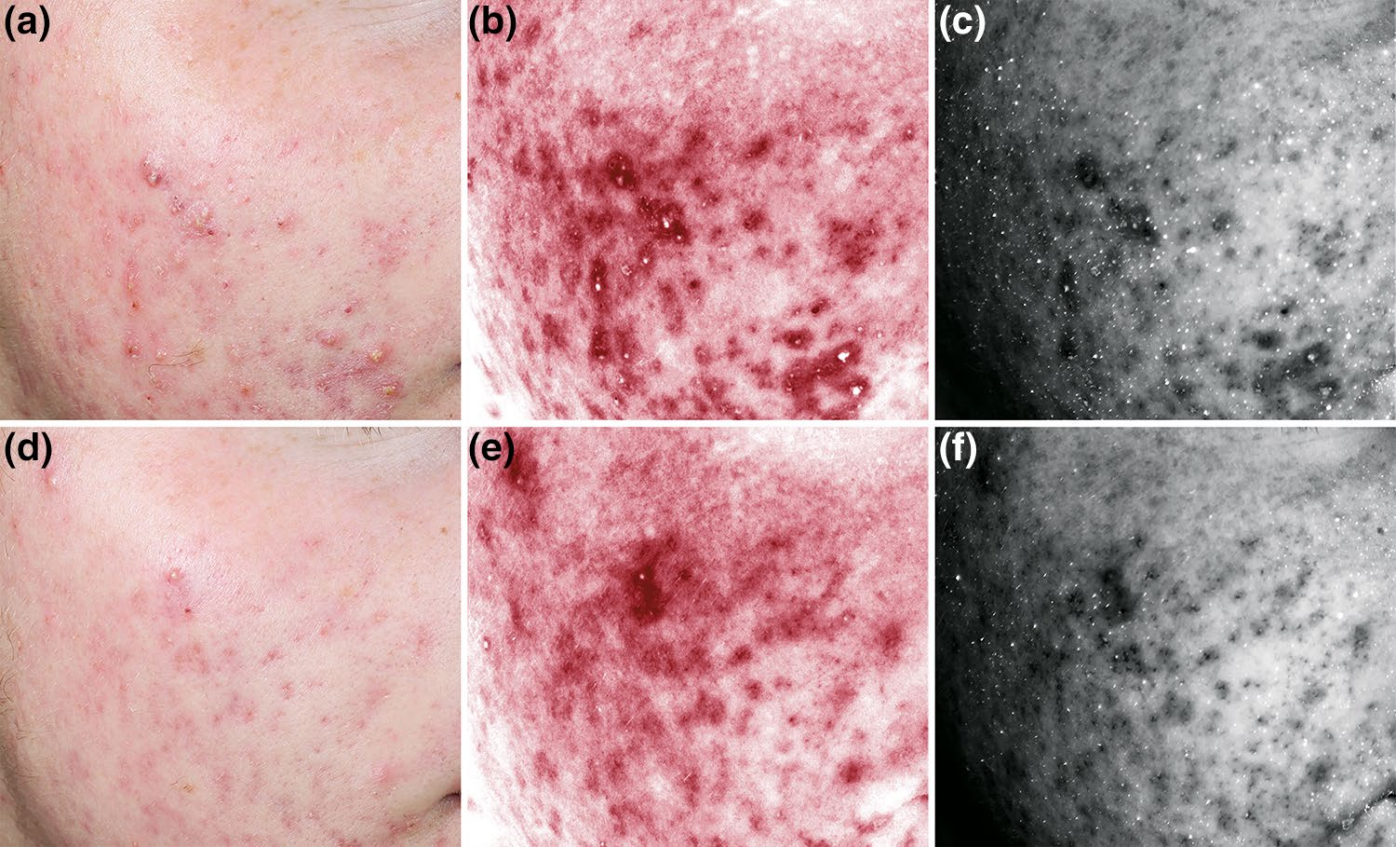
Change in acne severity and corresponding change in the CpIII fluorescence and acne-spots due to treatment: **a, d** Clinical, **b, e** RBX-Red, and **c, f** CpIII fluorescence cropped images showing a region of the subject’s face treated with the combination product Clindamycin + BPO at Baseline and Day 25

Figure 6 shows cropped Clinical, RBX-Red, and CpIII fluorescence images of a subject’s cheek area from the side treated with the combination product Clindamycin + BPO at Baseline and Day 25. Cropped higher resolution images are shown here to illustrate the clinical treatment effect and the associated change in the acne-specific inflammation (acne-spots) and CpIII fluorescence.

## Discussion

For decades, acne imaging research has focused on colour rather than the spectral nature of the porphyrin fluorescence signal [1, 9, 14]. The red fluorescence and green fluorescence seen in a UVA image have been generalized as porphyrin and horn fluorescence, respectively. VISIA-CR allows separation and measurement of CpIII and PpIX fluorescence signals when capturing full-facial images of acne subjects. In this study, for the first time, we have shown associations of CpIII fluorescence with comedonal lesions. An association between CpIII fluorescence and papulopustular lesions could not be observed because the fluorescence signal is strongly absorbed by the surrounding haemoglobin. However, an association of the lesion-specific inflammation (acne-spots) measurement obtained from RBX-Red images with the papulopustular lesions could be demonstrated. Our results suggest that these two measurements may provide specific assessments of acne severity.

We have evaluated the area distribution of the CpIII fluorescence signal as a fractional area measurement, as well as the total count, size, and intensity of the individual spots. The number of fluorescence spots filtered, based on their size, showed high correlations with the clinically visible comedones and justifies its use as the main feature in formulating a classification method reported earlier [16]. Even though the correlation coefficient was lower when comparing all of the detected fluorescence spots, a clear association between this measurement and clinical assessment was demonstrated. As evident from the treatment efficacy, this measurement may be more sensitive and specific in detecting varying effects based on different modes of treatment [2, 11, 12]. This may be attributed to the early indication of colonizing bacteria and treatment effect on emerging lesions. Since CpIII is produced by colonizing *P. acnes* bacteria, imaging and measuring this fluorescence signal should provide a severity indication of the clinically visible comedones as well as the pre-clinical microcomedones. Furthermore, if the treatment stops *P. acnes* bacterial growth/colonization, the reduced concentration of CpIII will reflect in the fluorescence signal and provide an efficacy measurement to the anti-microbial treatment.

Our results indicate a strong association between the investigators’ papulopustular lesion counts and the fractional area of acne-spots detected from the RBX-Red images. An auto-lesion classification system using this feature as one of the primary identifiers of papulopustular lesions has already been reported [16]. The acne-spot detector identifies circular haemoglobin concentrated areas and not the global redness or background redness due to treatment reaction. Although the detection may include both active and residual erythema, differentiating between the two is also challenging during clinical assessment. An anti-inflammatory treatment should prevent or reduce the number of comedones evolving into papulopustular lesions and reduce the inflammation of existing lesions. This treatment effect will reflect in the acne-spots fractional area measurement. Furthermore, since a self-evaluating patient is primarily focused on the acne-spots, this measurement may also relate better with patient-assessed outcomes. Using the fractional area of CpIII fluorescence spots and acne-spots as objective measures of comedonal and papulopustular lesion, severity, respectively, in interventional acne research can, therefore, be promising and beneficial.

Our data indicate weaker associations between spot size, location, and/or intensity of PpIX fluorescence signal with acne lesions. However, PpIX fluorescence also needs to be reevaluated in studies involving δ-aminolaevulinic acid (ALA) and the methyl ester of ALA (MAL). ALA or MLA taken up by *P. acnes* can result in an increased concentration of endogenous porphyrins, and in some colonies, a predominance of PpIX can be observed [8]. Furthermore, the facial distribution of PpIX mainly in areas with higher sebum production rate suggests a need to investigate the association between two.

In addition to providing indications of comedonal and papulopustular acne severity, measurement of the association between CpIII/PpIX and acne lesion-specific inflammation may be more sensitive and specific to treatment effects and the early signs of lesion progression. Being simple and objective, these quantitative measurements can be translated from clinical research to clinical practice. The higher sensitivity of these measurements in evaluating treatment efficacy can benefit phase-II dose-ranging clinical studies.
